# A Rare Case of Adult-onset Still’s Disease with Anti-Ro Antibody Positive

**DOI:** 10.7759/cureus.6055

**Published:** 2019-11-02

**Authors:** Soujanya Sodavarapu, Marium Asad, Rajani Rudrangi

**Affiliations:** 1 Internal Medicine, San Joaquin General Hospital, French Camp, USA; 2 Rheumatology, San Joaquin General Hospital, French Camp, USA

**Keywords:** adult onset still’s disease, anti-ro antibody, rash, lymphadenopathy, ana, fever, arthritis

## Abstract

Adult-onset Still’s disease (AOSD) is a rare systemic inflammatory disorder. The exact pathogenesis is unknown but believed to have multiple etiologies. The Yamaguchi criteria are used to aid in the diagnosis of AOSD. Typical characteristics are spiking fevers, febrile rash, arthritis, and the absence of other serologic markers of rheumatic diseases. We present a case of a 31-year-old Hispanic female who presented with fevers, arthritis, febrile rash, high ferritin levels, and cervical and axillary lymphadenopathies. The unique feature of our case is that the patient was positive for antinuclear antibody (ANA) titers of greater than 1:640 and anti-Ro antibody. She responded with the pulse dose steroids and later prescribed methotrexate and tapered off prednisone with improvement in her symptoms.

## Introduction

Adult-onset Still’s disease (AOSD) is a rare systemic inflammatory disorder with a yearly incidence of 0.16 per 100,000 adults [1]. Previously known as systemic juvenile idiopathic arthritis, it is an inflammatory disorder without known pathogenesis but believed to have multiple etiologies such as genetics and viral infections [2]. The main characteristics of the disease are spiking fevers, febrile rash, arthritis, and the absence of other serologic markers of rheumatic disease. It needs a high index of suspicion, and other conditions need to be excluded before diagnosing with AOSD.

## Case presentation

A 31-year-old Hispanic female presented to the ER with unresolved spiking fevers, generalized fatigue and weakness, and a sore throat, which began three weeks prior to presentation. She had also experienced a nonpruritic macular rash involving her trunk and upper extremities, which lasted a few hours before resolving on its own. She also reported suffering from diffuse joint pains and body aches, mainly in her wrists, hands, knees, ankles, and feet; these were associated with swelling and stiffness, which lasted all day. She denied experiencing any similar symptoms prior to the current episode. 

An examination of the patient's vital signs revealed a fever of 39.4°C and tachycardia with a heart rate of 123 beats per minute, but a normal respiratory rate and blood pressure. Cervical and axillary lymphadenopathies were also noted on physical examination. She had acute synovitis of both knees, both ankles, the right wrist, the third to fifth metacarpophalangeal joints in the right hand, and the first and second metacarpophalangeal joints and the proximal interphalangeal joints in the left hand. Her throat was mildly congested, but there were no other remarkable symptoms. 

**Figure 1:**
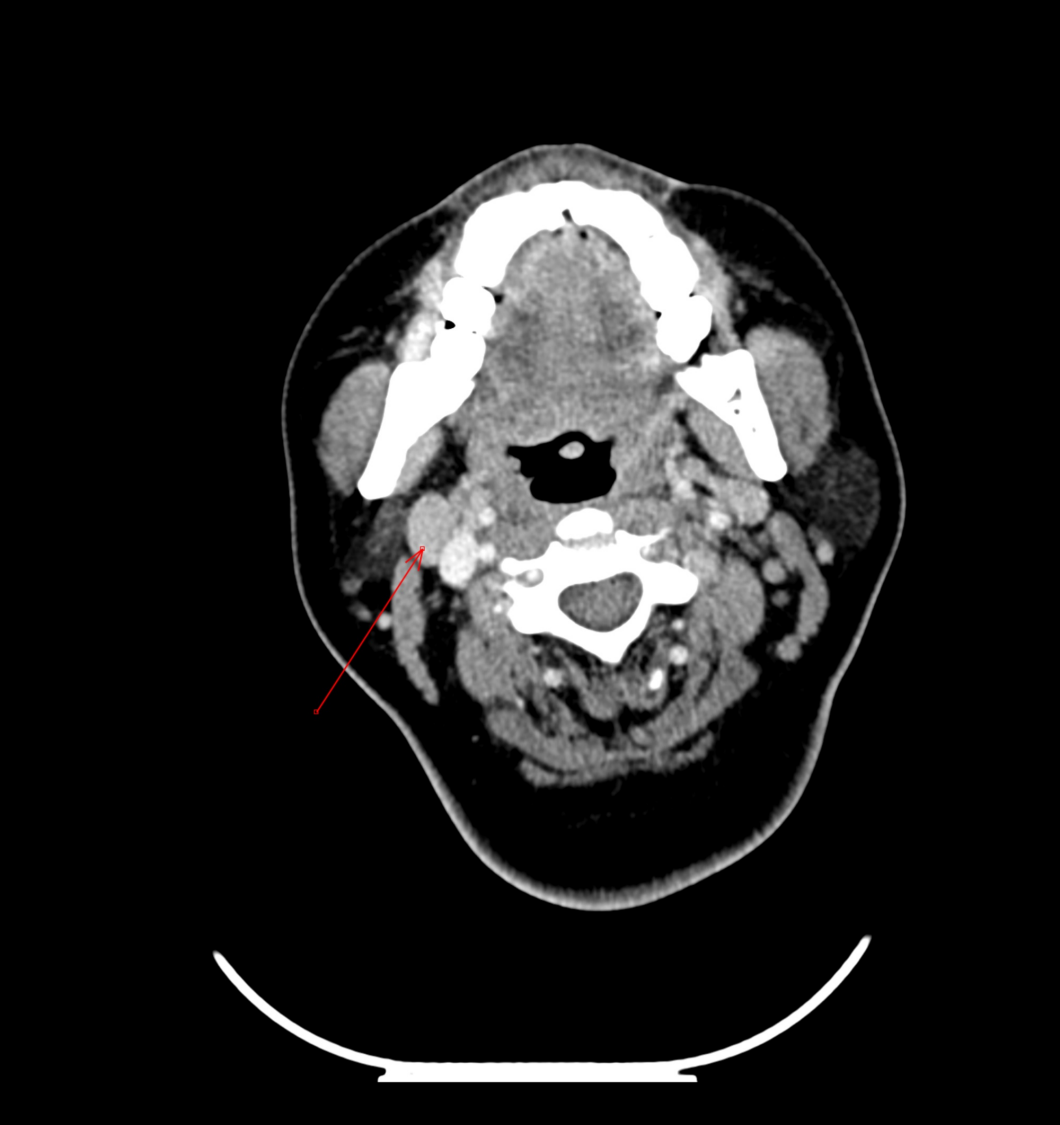
CT scan of the neck, arrow shows an enlarged cervical lymph node, anterior to the right jugular vein.

Laboratory investigations revealed an elevated leukocyte count of 17.6 × 109/L (90.0% neutrophils). Additionally, acute phase reactants were markedly elevated with an erythrocyte sedimentation rate (ESR) of 66 mm/h, a serum C-reactive protein (CRP) concentration of 29.38.4 mg/L, and a serum ferritin concentration higher than 40,000 µg/L. Moreover, she had an antinuclear antibody (ANA) titer higher than 1:640 and tested positive for anti-Sjögren's syndrome-related antigen A (SSA/Ro) antibodies. Conversely, she tested negative for rheumatoid factor (RF), as well as an anti-cyclic citrullinated peptide, anti-Smith (Sm), anti-Sm/ribonucleoprotein (Sm/RNP) antigen, and anti-La antibodies, with complement proteins complement 3 and complement 4 also being within normal limits. Additionally, liver, renal, and coagulation profiles were normal, while blood and urine cultures were both negative. Due to lymphadenopathy, tests for Coccidioides complement fixation, QuantiFERON-TB Gold, HIV, hepatitis C antibody, and hepatitis B surface antigen were done, with all results being negative. CT scans of the patient's neck and chest revealed cervical and axillary lymphadenopathies, but the lesions were too small for biopsy (Figure 1). CT scans of the abdomen/pelvis and positron emission tomography (PET) scans were unremarkable. Likewise, a bone marrow biopsy was negative for malignancy.

Therefore, our patient met the Yamaguchi criteria based on her clinical features and laboratory investigations and was diagnosed with AOSD. She was started on pulse dose steroids with IV methylprednisone (125 mg every 8 h), became febrile in 48 h with symptom improvement, and was discharged home on oral prednisone (60 mg daily). However, she was readmitted seven days later for fever and joint pains. She was given pulse dose steroids again with IV methylprednisone (50 mg every 6 h) and reported improvement in her symptoms after three days. She was then discharged on oral prednisone (60 mg daily) with daily supplementation of calcium and vitamin D. Initially, she had elevated levels of liver enzymes, so methotrexate was not started immediately. One month later, her liver function tests improved, and she was started on methotrexate (10 mg weekly). During the subsequent follow-up, her symptoms improved, and her steroids were tapered off.

## Discussion

Adult-onset Still’s disease is a rare systemic inflammatory disorder. It has multiple etiologies, such as genetics and viral infections [2]. Examples of viral infections related to AOSD include Rubella, echovirus 7, and Parvovirus B19 [3-5]. Patients with AOSD usually present with fever, arthritis, and rash [6]. Temperatures are typically high grade and occur mainly in the evening [7]. The rash that occurs in AOSD is usually salmon-colored, maculopapular, involving the trunk and upper extremities, and primarily occurs with the onset of fever [6]. Arthritis and arthralgia in AOSD involve mainly the knees, ankles, and wrists [6]. Less common signs of AOSD include hepatomegaly, abdominal pain, pericarditis, and pleurisy [6]. Our patient’s presentation was consistent with findings of AOSD as she had a three-week history of high-grade fever, arthralgias, intermittent nonpruritic rash, sore throat, and lymphadenopathy.

Diagnostic workup for AOSD may include laboratory tests, diagnostic imaging, and biopsy of skin or lymph nodes. Typical laboratory findings include elevated ESR and CRP, leukocytosis, and high serum ferritin [8]. Markedly elevated serum ferritin (values exceeding 3000 ng/mL) is a hallmark feature of AOSD [9]. Other associated laboratory studies may show normocytic normochromic anemia and leukocytosis with a neutrophil predominance [7].

Surprisingly, immunologic studies such as ANA and RF are usually present in only 10% of patients with AOSD. If present, they are typically low in titers [6]. The Yamaguchi criteria, founded in 1992, are widely used to aid in the diagnosis of AOSD as they have 96% sensitivity and 92% specificity [10]. Yamaguchi criteria entail four major and four minor criteria out of which patients need to meet five features, with at least two being major criteria, to be diagnosed with AOSD (Table 1).

**Table 1:** Yamaguchi criteria needed to diagnose AOSD. Patient must have five features, with at least two being major criteria, to be diagnosed with AOSD. AOSD: Adult-onset Still's disease

Yamaguchi criteria	
Major criteria	Minor criteria
Fever > 39°C for at least one week	Sore throat
Arthralgia or arthritis for at least two weeks	Lymphadenopathy
Typical rash	Hepatomegaly or splenomegaly
Leukocytosis of at least 10,000 (>80% neutrophils)	Abnormal liver function tests
	Negative antinuclear antibody and rheumatoid factor

Our patient met the Yamaguchi criteria, given that she had spiking fevers, nonpruritic macular rash, arthritis, lymphadenopathy, and leukocytosis. Our patient also had an outstanding serum ferritin level of approximately 40,000. However, what stands out is that our patient was also positive for ANA and anti-Ro antibody, an antibody commonly found positive in Sjögren's disease. As mentioned above, immunologic markers are present in less than 10% of patients diagnosed with AOSD. Upon literature review, a case report was found where an infant patient diagnosed with neonatal lupus (NL) at birth was later found to have juvenile idiopathic arthritis at three years of age [11]. NL occurs when there is transplacental migration of Anti-Ro and Anti-La antibodies to the fetus [11].

Standard goals of therapy for the treatment of AOSD include: 1) treating signs and symptoms, 2) preventing end-organ damage, and 3) limiting side effects of therapy drugs [12]. In patients with minimal symptoms, nonsteroidal anti-inflammatory drugs (NSAIDs) relieve symptoms in approximately 20% of patients [13]. In patients with moderate disease, glucocorticoids are the treatment of choice, with prednisone at a dose of 0.5-1 mg/kg, as the preferred steroid [8, 12]. Ideally, steroids are tapered off; however, some patients become glucocorticoid dependent on controlling their symptoms. For those patients, biologics or disease-modifying antirheumatic drugs (DMARDs) are initiated.

Our patient was initially given pulse dose IV methylprednisone and discharged on oral prednisone therapy, but she did not respond and continued to have symptoms. Therefore, pulse steroid therapy was attempted again with IV methylprednisone for three days, this time with an adequate response. Our patient was subsequently started on methotrexate-prednisone dual treatment, followed by a gradual taper of prednisone, and is now tolerating the treatment well.

## Conclusions

Adult-onset Still’s disease diagnosis needs a high index of suspicion. In patients presenting with rash, arthritis, and fever, it should be considered after ruling out possible infections, malignancies, and other rheumatologic diseases. Surprisingly immunologic factors are present in less than 10% of AOSD. There have been case reports with ANA and RF positivity, but none reported with an anti-Ro antibody. To our knowledge, this is the first case report of AOSD with a high ANA titer and positive testing for anti-Ro antibodies.
